# Style-Enhanced Transformer for Image Captioning in Construction Scenes

**DOI:** 10.3390/e26030224

**Published:** 2024-03-01

**Authors:** Kani Song, Linlin Chen, Hengyou Wang

**Affiliations:** School of Science, Beijing University of Civil Engineering and Architecture, Beijing 100044, China; 15083122562@163.com (K.S.); chenlinlin@bucea.edu.cn (L.C.)

**Keywords:** image captioning, construction scene, style feature, transformer

## Abstract

Image captioning is important for improving the intelligence of construction projects and assisting managers in mastering construction site activities. However, there are few image-captioning models for construction scenes at present, and the existing methods do not perform well in complex construction scenes. According to the characteristics of construction scenes, we label a text description dataset based on the MOCS dataset and propose a style-enhanced Transformer for image captioning in construction scenes, simply called SETCAP. Specifically, we extract the grid features using the Swin Transformer. Then, to enhance the style information, we not only use the grid features as the initial detail semantic features but also extract style information by style encoder. In addition, in the decoder, we integrate the style information into the text features. The interaction between the image semantic information and the text features is carried out to generate content-appropriate sentences word by word. Finally, we add the sentence style loss into the total loss function to make the style of generated sentences closer to the training set. The experimental results show that the proposed method achieves encouraging results on both the MSCOCO and the MOCS datasets. In particular, SETCAP outperforms state-of-the-art methods by 4.2% CIDEr scores on the MOCS dataset and 3.9% CIDEr scores on the MSCOCO dataset, respectively.

## 1. Introduction

Image captioning [1,2,3] is one of the fundamental problems in the intersection of computer vision (CV) and natural language processing (NLP). Its main task is to understand the context of the image and interpret it with natural language to realize the mutual mapping between image features and text features. With the commercialization of sensor networks and IoT technologies, the amount of data available in the construction industry continues to increase. Describing the semantic content of images in construction scenes can help managers quickly grasp the information of construction activities, make timely decisions, and improve the degree of automation and intelligence of project management. Therefore, image captioning in construction scenes has become an important research issue in intelligent construction.

There have been many excellent studies in the field of image captioning. One of the important research directions is multimodal learning, which is the joint modeling of image and text information. One common strategy is to use an attention mechanism that allows the model [4,5] to selectively focus on different areas or features in the image to generate more accurate, detailed descriptions. In addition to the traditional methods based on supervised learning, there have been some studies on unsupervised learning and self-supervised learning in recent years. These methods [6,7] work by leveraging large amounts of unlabeled image and text data from which they learn the correspondence between images and text, as well as the ability to generate descriptions. In addition, some studies [8,9] have focused on solving the problems of long text generation and diversity. Long text generation aims to generate longer, more detailed descriptions, while diversity aims to make the generated results richer and more varied, avoiding repetition and drudgery. However, these methods have achieved satisfactory results in public datasets, such as MSCOCO [10], but they are not suitable for the complex environments of construction scenes.

Previous research primarily focused on target-detection tasks based on construction scene images to address the intelligent management of construction scenes. Some studies [11,12,13,14] using target-detection methods have addressed safety-related issues, such as determining safety levels or detecting the presence of safety equipment. However, these studies only use visual information, ignoring the large amount of available textual information. More recently, some researchers have started to focus on image captioning in construction scenes and carry out targeted research based on the characteristics of construction scenes. For example, Bang et al. [15] used a dense network to describe the construction images collected by drones or other video capture equipment. Liu et al. [16] proposed an encoder-decoder framework to describe five types of construction activities. Han et al. [17] constructed their model for the construction scenes by replacing the professional vocabulary. However, the extant studies are limited by certain problems, including less variety of construction activities under research, inadequate consideration of the size and stylistic features of datasets, as well as suboptimal model performance. To solve the above problems, we propose improvements from three aspects: model architecture, model training, and loss function to improve the performance of the image-captioning model.

In terms of model architecture, previous approaches to image captioning have predominantly relied on convolutional neural networks (CNN) and long short-term memory networks (LSTM). CNN is used for encoding spatial features, while LSTM decodes these features into textual descriptions, achieving certain success in image-captioning tasks [18,19,20] on public datasets. However, these architectures are limited by the expressive power and training efficiency of LSTM and cannot further improve their performance. Recently, Transformer [21] models have been proven to be more effective in capturing long-range dependencies in data and achieving superior performance on public datasets. Therefore, we will propose an improved framework based on Transformer.

**Figure 1:**
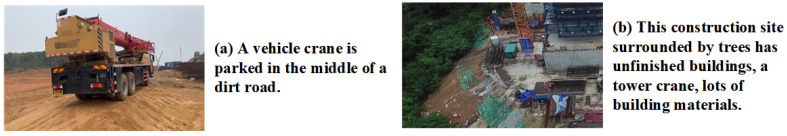
This is a presentation of two styles of images. Picture (**a**) contains fewer targets and can clearly describe the target’s ongoing activities; Picture (**b**) contains a large number of targets, so it is difficult to describe the activities of each target in detail in one sentence, so we use one sentence to summarize the targets contained in the picture.

Although some Transformer-based studies [11,22] have demonstrated outstanding performance on the MSCOCO dataset, they have not taken into account the visual style of construction site images, hindering their adaptability to construction scenes. The visual style of construction site images is one of the important factors in the study of image captioning, which can influence the direction of semantic descriptions. Specifically, as depicted in Figure 1a, when an image contains few objects and a relatively simple environment, we are more likely to use <subject-verb-object> formats to describe specific objects. Conversely, Figure 1b illustrates that we are more likely to list all objects in the image if it contains numerous objects and a complex environment. Thus, to consider the visual style inherent to construction scenes, we will propose a novel model framework. In particular, due to its excellent performance in feature extraction, we use the Swin Transformer [23] as the backbone to extract grid features, which are subsequently initialized and transformed as semantic and style features. Then, two more refined encoder layers based on Transformers are constructed to encode the semantic features and style features individually. The encoded style feature is employed as supplementary input alongside the reference sentences to the decoder. Within the decoding layer, the text features of reference sentences interact with semantic features to generate the final captions.

In terms of model training, we conduct our research from two aspects: data preparation and training mode. For data preparation, due to the lack of publicly available text description datasets specific to construction scenes, we construct a text description dataset for training based on the MOCS [24] dataset. Because of the high costs of text annotation, we only annotate 5000 images in the dataset. Meanwhile, we enhance the training data to improve the performance of the model. For training mode, owing to huge stylistic disparities between images in public datasets and those from construction scenes, the direct application of a model [25,26,27] exhibiting exceptional performance on public datasets to construction scenes datasets will not yield ideal results. In addition, given the inherent challenges associated with training a stable model using a small dataset, this study adopts a two-step approach, including pre-training and fine-tuning. Initially, the model is trained on a public training set to enhance its interactive capabilities, perceptual acuity, and overall stability. Subsequently, fine-tuning is undertaken using the construction site training set to refine the model’s applicability within the context of construction scenes.

In terms of the loss function, we add sentence style loss to the common cross-entropy loss to optimize the model’s performance. Different training sets have different labeling styles. For example, each dataset has its own attributes in terms of sentence length and parts-of-speech order. We introduce sentence style loss to make the model learn the style change in the dataset when migrating from the public dataset to the construct scenes dataset.

In summary, the main contributions of our research include:

1. To enhance the style of the generated sentence, we propose a novel model framework to encode semantic features and style features, respectively. Then, we integrate style features into text features to guide the decoder to generate sentences word-for-word. Finally, we add the sentence style loss into the total loss function to make the style of the generated sentences more biased towards the MOCS training set.

2. To establish an image-captioning dataset specifically tailored to construction site scenes, we label a text description dataset based on the MOCS. At the same time, due to the limited scale of the labeled dataset, augmentation techniques for the data are employed to enhance the robustness of model training.

3. To transition from public datasets to the construction scenes dataset, we employ a pre-training and fine-tuning paradigm, effectively enabling the model to achieve exceptional performance even when confronted with small-scale sample datasets.

The proposed model framework achieves excellent results on both the MSCOCO dataset and the MOCS dataset. These research findings are not only significant for improving image captioning in construction scenes but also provide valuable insights and inspiration for image-captioning research in other domains.

## 2. Related Work

In recent years, the field of image captioning has undergone rapid development in terms of model frameworks and application scenarios. For model frameworks, the effectiveness of Transformer models in capturing long-range dependencies in sequence modeling has been widely acknowledged, leading to the development of various image-captioning models based on Transformer architectures. In terms of application scenarios, studies on image captioning in construction scenes remain relatively scarce, and more research can be done. In this section, we briefly review some related work about image captioning based on Transformer and model applications in construction scenes.

### 2.1. Transformer-Based Models

Currently, numerous researchers have proposed remarkable accomplishments in the domain of image captioning by leveraging the Transformer framework [5,28,29]. For instance, Huang et al. [22] proposed an Attention-on-Attention module to establish correlations between attention results and queries. Additionally, Kim et al. [11] developed a pure vision Transformer model that uses grids to represent features without extracting regional characteristics. An end-to-end image-captioning framework has also been proposed that utilizes Transformer models to capture factual information and style elements within images to generate stylized descriptions [30]. While such models have shown impressive performance on public datasets, they do not fully account for the impact of complex environmental conditions or non-daily activities on results. Thus, they cannot be directly applied to describe images in construction scenes.

### 2.2. Model Application in Construction Scenes

In recent years, there has been some work on the image captioning of construction scenes. Bang et al. [15] used a dense caption network to describe the images captured by drones at construction sites. However, this method only focuses on the local information of the target area in the construction images and cannot capture and perceive the style information of the construction scenes, so the text description of the style information of the construction scenes cannot be generated. Liu et al. [16] studied construction activities using image captioning and were able to generate text descriptions related to activities of construction scenes. However, there are limitations to studying a limited number of construction activities. Nong et al. [31] employed an attention mechanism and the encoding-decoding architecture to describe the semantic content of construction scenes. This approach demonstrated the ability to generate accurate descriptions even in challenging conditions such as low lighting, night-time operations, and the presence of distant and densely distributed targets. However, the study failed to consider the stylistic aspects of the generated sentences and the use of a relatively small dataset.

## 3. Model

**Figure 2:**
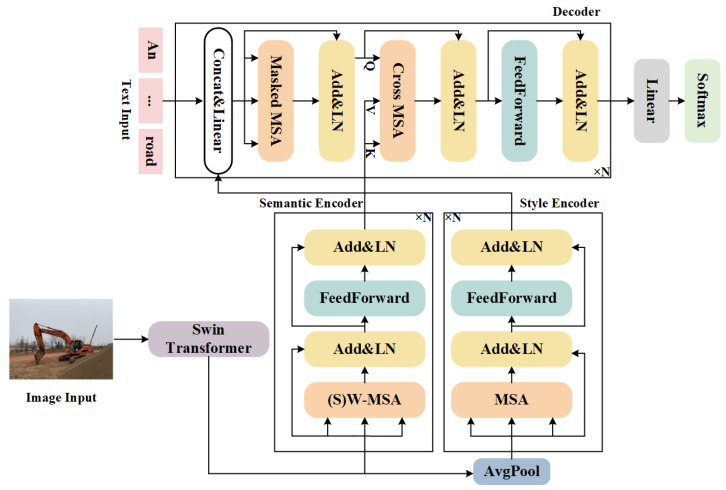
The overall framework of our model. First, we use the Swin Transformer to extract grid features. Second, we initialize grid features as semantic features and style features and then send them to the detail semantic encoder and style encoder, respectively. Subsequently, the input text features first to fuse the style feature and then interact with the semantic features in the decoder. Finally, we use Softmax to obtain the probability distribution of the model about the vocabulary and obtain the description sentence.

To generate image descriptions under construction scenes, a style-enhanced Transformer for image captioning, which is simply called SETCAP, is proposed. The framework of the forward reasoning model is shown in Figure 2. The whole framework adopts the commonly used encoder-decoder structure. The encoder consists of *N* semantic encoding blocks and style encoding blocks based on the Transformer encoding layer. The decoder consists of *N* Transformer decoding blocks. Swin Transformer is responsible for extracting the grid features of the image. The encoder layer senses the semantic content of the image from both the semantic details and the style features, and the decoder layer captures the semantic connection between the image and the text and generates the image description word by word.

### 3.1. Feature Extraction

Given the complexity of construction scenes and large differences in object scale, inspired by [32,33], this study chooses to explore grid features. Swin Transformer extracts features by splitting the image into non-overlapping small pieces and cross-layer communication among the different layers of small pieces. This feature extraction method is helpful to capture global semantic features and improve the model’s understanding of the overall image structure. Therefore, using the Swin Transformer can help enhance the detection and recognition of multi-scale objects.

After the Swin Transformer is used to extract image features, a linear function maps the features to the dimensions required by the Transformer. In this case, the obtained features can be regarded as semantic features rich in semantic meaning, and the semantic features are initialized into style features through global average pooling. We encode the semantic feature and style feature, respectively, through the semantic encoder module and the style encoder module.

### 3.2. Encoder

For a given image *I*, we use Swin Transformer as the backbone to extract grid features $V_{grid}=\left\{v_{1},v_{2},\ldots ,v_{m}\right\}$, $v_{i}\in R^{D}$, where *D* represents the embedded dimension of each grid feature, and *m* denotes the number of grid features. We built two more detailed encoders to capture the semantic and style information and understand the deeper connections of objects within the image.

#### 3.2.1. Style Encoder

The grid features are transformed into the initial value of style features $V_{st}$ after average pooling. Then, the initialed style features are sent to the style encoder containing the MSA module.

MSA. The multi-head self-attention mechanism extends the attention mechanism to multiple parts, thus enhancing the model’s attention to different aspects of features. It effectively improves the model’s expressiveness and promotes the model to learn more complex and diversified features. To calculate the MSA score, we first divide the input into multiple heads, calculate the attention score of each head, and concatenate the calculation results to obtain the final calculation result:

(1) $$Attention\left(Q,K,V\right)=Softmax\left(\frac{QK^{⊤}}{\sqrt{d}}\right)V,$$

(2) $$head_{i}=Attention\left(Q^{i},K^{i},V^{i}\right),i=1,2,\ldots ,h,$$

(3) $$MSA\left(Q,K,V\right)=Concat\left(head_{1},\ldots ,head_{h}\right).$$

where $Q^{i},K^{i},V^{i}$ is the *i*-th slice of Q,K,V, and $Q,K,V\in R^{m\times D}$,$Q^{i},K^{i},V^{i}\in R^{m\times \frac{D}{h}}$. In the encoder, the Q,K,V input of the multi-head self-attention mechanism is all about image information. We use *Q* and *K* to calculate the similarity, which is regarded as the weight, and calculate the weighted sum of *V*. Therefore, the multi-head self-attention mechanism in the encoder can capture the internal connection of the image information.

FeedForward. The style encoder consists of *N* layers stacked in sequence. Layer *l* can be represented as:

(4) $$\begin{array}{c} {\hat{V}}_{st}^{l}=LayerNorm\left(V_{st}^{l-1}+MSA\left(W_{TQ}^{l}V_{st}^{l-1},W_{TK}^{l}V_{st}^{l-1},W_{TV}^{l}V_{st}^{l-1}\right)\right), \end{array}$$

(5) $$V_{st}^{l}=LayerNorm\left({\hat{V}}_{st}^{l}+FeedForward\left({\hat{V}}_{st}^{l}\right)\right).$$

where $V_{st}^{l-1}$ represents the style features of the output of layer l−1 and is used as input to layer *l*. $V_{st}^{0}=V_{st}$, and $W_{TQ}^{l},W_{TK}^{l},W_{TV}^{l}$ are the parameter matrices that need to be learned. The FeedForward function consists of two linear layers, using the ReLU activation function, and is represented as:

(6) $$FeedForward\left(x\right)=W_{2}ReLU\left(W_{1}x\right),$$

where $W_{1},W_{2}$ are the parameter matrices. The output of the encoding layer *N* is merged into text features.

#### 3.2.2. Semantic Encoder

**Figure 3:**
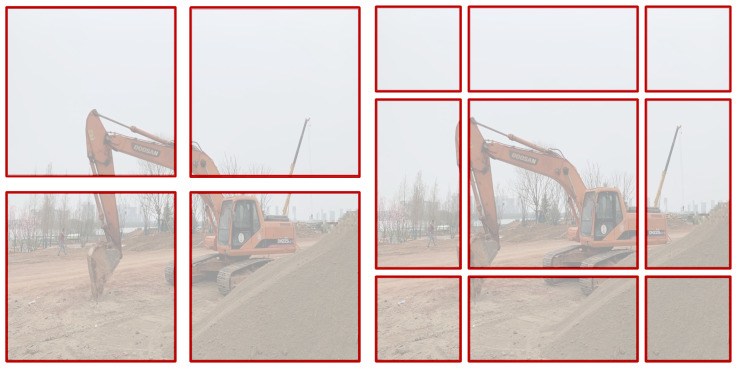
Method of dividing regular window and shifted window.

We take grid features as the initial value of semantic features $V_{se}$, and semantic features are sent to the semantic encoder containing the W-MSA or SW-MSA module.

(S)W-MSA. MSA has high computational complexity. To solve this problem, Swin Transformer proposes W-MSA and SW-MSA to calculate self-attention in a local window, which saves a lot of computation compared with the MSA module. The semantic encoder uses W-MSA and SW-MSA modules to calculate attention scores, where the inputs of *Q*, *K*, and *V* are all about the image information and have the same length *L* and dimension *D*. W-MSA and SW-MSA first divide the input of *Q*, *K*, and *V* into multiple windows and then apply MSA to each of the multiple windows. W-MSA cannot transfer information between windows, so SW-MSA is added after W-MSA to solve the problem of insufficient cross-window connection and further improve the modeling capability. The following Figure 3 shows the conventional window partitioning method and the shifted window partitioning method.

The modeling process of W-MSA and SW-MSA can be represented as follows:

(7) $$window_{i}=MSA\left(Q^{W_{i}},K^{W_{i}},V^{W_{i}}\right),i=1,2,\ldots ,w,$$

(8) $$\left(S\right)W-MSA=Merge(window_{1},\ldots ,window_{w}).$$

where *w* represents the number of windows, $Q^{W_{i}},K^{W_{i}},V^{W_{i}}$ represent the slice under the *i*-th window. Merge is the inverse operation of dividing regular windows and shifted windows. Through W-MSA and SW-MSA, the model can perceive complex environments and objects of varying sizes in images. The semantic encoder consists of modules including the FeedForward layer, W-MSA, or SW-MSA. SW-MSA is used after W-MSA, and the two are alternately used.

FeedForward. The semantic encoder also consists of *N* blocks stacked in sequence. Take the *l* layer encoder as an example:

(9) $$\begin{array}{c} {\hat{V}}_{se}^{l}=LayerNorm(V_{se}^{l-1}+\left(S\right)W-MSA\left(W_{EQ}^{l}V_{se}^{l-1},W_{EK}^{l}V_{se}^{l-1},W_{EV}^{l}V_{se}^{l-1}\right), \end{array}$$

(10) $$V_{se}^{l}=LayerNorm\left({\hat{V}}_{se}^{l}+FeedForward\left({\hat{V}}_{se}^{l}\right)\right).$$

where $V_{se}^{l-1}$ represents the semantic features of the output of layer l−1 and is used as input to layer *l*. $V_{se}^{0}=V_{se}$, and $W_{EQ}^{l},W_{EK}^{l},W_{EV}^{l}$ are the parameter matrices. The FeedForward function also consists of two linear layers and the ReLU activation function:

(11) $$FeedForward\left(x\right)=W_{4}ReLU\left(W_{3}x\right)$$

$W_{3},W_{4}$ are the parameter matrices. The output of the encoding layer *N* is used as the input of the decoder.

### 3.3. Decoder

The goal of the decoder is to interact and map the visual features of the encoder and the textual features of the decoder input, generating descriptions word by word. As shown in Figure 2, the decoder consists of *N* sequentially stacked blocks, each consisting of three parts: feature prefusion, language-masked MSA, and feature interaction. The initial input to the first layer of the decoder is the mapping vector $X^{0}$ of the reference sentence words, where *x* is the one-hot encoding of the reference sentence words and $W_{e}$ is the word mapping matrix:

(12) $$X^{0}=W_{e}x.$$

$X^{l-1}$ is the output of l−1 layer. Take the *l*-layer decoder as an example. In the feature prefusion module, the output $v_{st}^{N}$ of the last layer of the style encoder is fused with the text features as auxiliary information. Specifically, $v_{st}^{N}$ concatenates text embedding features $X^{l-1}$ and multiplies a weight matrix $W_{g}$:

(13) $$X_{concat}^{l}=W_{g}\left[v_{st}^{N},X^{l-1}\right].$$

$X_{concat}^{l}$ is the result of the fusion of image style information and text embedding information and is the input of the language-masked MSA module. The language-masked MSA module calculates attention scores of $X_{concat}^{l}$ to capture the logic inherent in the text features to model the internal modal relationships, which is expressed as:

(14) $$\begin{array}{c} X_{masked}^{l}=LayerNorm\left(X_{concat}^{l}+MSA\left(W_{Q}^{m,l}X_{concat}^{l},W_{K}^{m,l}X_{concat}^{l},W_{V}^{m,l}X_{concat}^{l}\right)\right), \end{array}$$

where $W_{Q}^{m,l},W_{K}^{m,l},W_{V}^{m,l}$ are the parameters matrices. In the feature interaction module, text features $X_{masked}^{l}$ and image semantic features $V_{se}^{N}$ carry out feature interaction in the next MSA module, calculate the similarity degree of text features and image features, and realize the mutual mapping between image features and text features. The process is expressed as follows:

(15) $$\begin{array}{c} X_{cross}^{l}=LayerNorm\left(X_{masked}^{l}+MSA\left(W_{Q}^{c,l}X_{masked}^{l},W_{K}^{c,l}V_{se}^{N},W_{V}^{c,l}V_{se}^{N}\right)\right), \end{array}$$

(16) $$X^{l}=LayerNorm\left(X_{cross}^{l}+FeedForward\left(X_{cross}^{l}\right)\right).$$

where $W_{Q}^{c,l},W_{K}^{c,l},W_{K}^{c,l}$ are parameter matrices. $X_{cross}^{l}$ is decoded to obtain the expression result $X^{l}$, which integrates image semantic features, style information, and text features. After *N*-layer decoding, the final result $X^{N}$ is obtained. $X^{N}$ is sent into the Softmax function to obtain the probability distribution of the model about the entire vocabulary:

(17) $$p\left(x\right)=Softmax\left(W_{x}X^{N}\right).$$

$W_{x}$ is the parameter matrix that needs to be learned.

## 4. Loss Function

The last layer output of the decoder is passed through the Softmax function to obtain the probability distribution of the model about the entire vocabulary. The target of training is to generate a sentence similar to the reference sentence. Thus, we need to optimize our model by calculating the generated probability distribution and the cross-entropy loss:

(18) $$L_{caption}\left(\theta \right)=-\sum_{t=1}^{T}log\left(p_{\theta }\left(y_{t}^{*}|y_{1:t-1}^{*}\right)\right),$$

where $y_{1:T}^{*}$ represents the standard reference sentence and θ represents the parameters of the model. Since the number of MSCOCO training sets is larger than the number of construction site data sets we constructed, pre-training on MSCOCO data sets can improve indicators, but it will also affect the sentence style of description sentences, i.e., in the process of domain adaptation, it is necessary to consider the conversion from the source domain style to the target domain style. In this case, the source domain is the MSCOCO dataset, and the target domain is the MOCS dataset. To make the generated sentences more similar to the target domain style, a sentence style loss is added:

(19) $$L_{style}\left(\theta \right)=-\sum_{t=1}^{T}log\left(p_{\theta }\left(s_{t}^{*}|s_{1:t-1}^{*}\right)\right),$$

where *s* is the property of the generated word at the time step *t*. The sentence style loss is multiplied by an adaptive parameter, and the total loss is the weighted sum of two cross-entropy losses:

(20) $$L_{XE}\left(\theta \right)=L_{caption}\left(\theta \right)+\lambda *L_{style}\left(\theta \right).$$

After using XE loss to optimize the model, the self-critical sequential training (SCST) [34] strategy is adopted to optimize the CIDEr index:

(21) $$L_{R}\left(\theta \right)=-E_{y_{1:T}∼p_{\theta }}\left[r\left(y_{1:T}\right)\right],$$

in the equation, r(·) represents the CIDEr score. An approximation of the gradient for $L_{R}$ can be expressed as follows:

(22) $$\nabla_{\theta }L_{R}\left(\theta \right)\approx -(r\left(y_{1:T}^{s}-r\left({\hat{y}}_{1:T}\right)\right)\nabla_{\theta }logp_{\theta }\left(y_{1:T}^{s}\right),$$

$y_{1:T}^{s}$ is the sentence obtained from sampling, and ${\hat{y}}_{1:T}$ is the sentence obtained from greedy decoding.

## 5. Dataset

### 5.1. Image Captioning Dataset

For most image-captioning tasks, public datasets such as MSCOCO are often used. However, the images included in this open dataset are mostly scenes of daily life, which are quite different from the scene activities of construction sites. Therefore, only using open datasets cannot meet the needs of training image caption tasks for construction scenes. However, there is no public text description dataset for construction scenes at present, so it is necessary to build an image-captioning dataset based on construction scenes.

**Figure 4:**
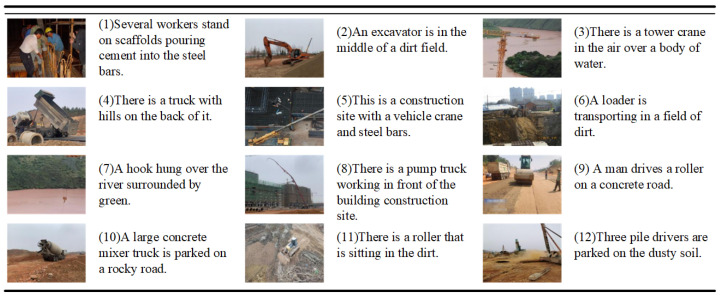
Partial image-captioning examples are labeled by our method on dataset MOCS.

To generate the image description for the construction scenes, we use the public construction scenes image dataset MOCS as the foundation and select 5000 images from the MOCS dataset to annotate with a description sentence according to the specific scene content. Some examples of description sentences are shown in Figure 4. The constructed dataset covers 12 kinds of common objects, reflecting the construction site’s real situation. A total of 60% of the image-text pairs in the dataset are randomly selected as the training set, comprising 3000 image-text pairs. The remaining 40% are selected as the test set to verify the training effect of the model. Due to the limited amount of training data, the model is initially pre-trained on the MSCOCO dataset. MSCOCO contains 123,287 images, each with five reference captions. According to the Karpathy segmentation setting [35], 113,287 images are used for training, 5000 for verification, and 5000 for testing.

To build MSCOCO’s vocabulary, we convert all words to lowercase, filter out words that occur less than six times, and collect the remaining 9487 words to build the vocabulary. Moreover, the description of the construction scenes contains industry-specific terms that are not covered by the original MSCOCO vocabulary. Thus, to extend MSCOCO’s vocabulary, we convert all words of MOCS to lowercase, filter out words that are not part of the MSCOCO vocabulary, and add them to the MSCOCO vocabulary. This allows the final vocabulary to contain the technical terms required for the fine-tuning phase, ensuring continuity between the pre-training phase and the fine-tuning phase. The final vocabulary contains 9762 words.

### 5.2. Data Enhancement

**Figure 5:**
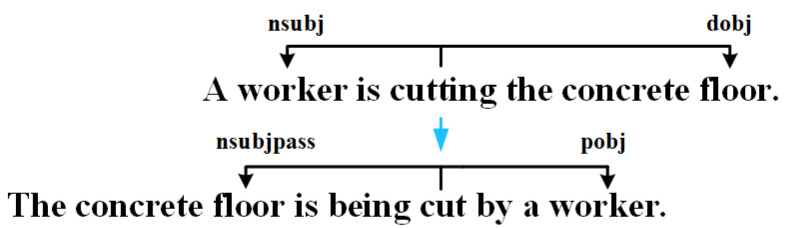
This is an example of data enhancement. Replace the object and subject of the sentence and change the tense of the sentence.

Due to the small amount of training data, we perform data enhancement to improve the robustness of model training. Referring to the idea of [36], based on the dependency tree of a sentence, the sentence is transformed according to the rules. For example, the subject and object of the original sentence are exchanged. And the sentence is changed from the active voice to the passive voice. As shown in Figure 5, by changing a sentence from the active voice to the passive voice, the worker is changed from the subject to the object and the floor changes from the object to the subject. Compared with the original text, the augmented data generated by sentence transformation at the sentence level has noise related to sentence patterns, which enhances the complexity of the text and makes the model more robust while keeping the semantics of the data unchanged.

We perform data enhancement for each training sentence, expanding the training set from 3000 to 6000, i.e., there are two reference sentences for each picture.

## 6. Experiment and Result

### 6.1. Parameter Settings

We set the number of encoder and decoder layers *N* as 3, the model embedding size *D* as 512, and the number of Transformer heads to 8. Set the epoch to 20 times when training the model with cross-entropy loss and 30 times when optimizing the model with SCST. Both of the above stages use the Adam [37] optimizer. The beam size is set to 5 during the evaluation phase.

### 6.2. Pre-Training

Due to the limited amount of training data in the MOCS dataset, our model cannot be directly trained on it. Additionally, we also lack other public captioned datasets related to construction scenes for model training. Therefore, in this work, to address this issue, we pre-train the model using the MSCOCO dataset and then fine-tune it based on the MOCS dataset to better capture the characteristics of construction scenes, resulting in more comprehensive training. Although the MSCOCO dataset does not include images of construction scenes, it can provide a substantial amount of training data that enables the model to learn the mapping from images to texts. This is similar to Transfer Learning.

The process of pre-training can capture the process of model learning from public datasets to image-to-text transformation, as well as the general word-order logic of text description. After the completion of pre-training, fine-tuning by the MOCS dataset can learn the unique sentence style of the MOCS dataset, as well as the mode of image-to-text transformation in the construction scenes. The training parameters of the two stages are consistent, and the training parameters are shown in the previous section.

### 6.3. Evaluation Metrics

In the evaluation phase, we evaluate the quality of the image description using five established metrics: BLEU [38], METEOR [39], ROUGE_L [40], CIDEr [41], and SPICE [42]. BLEU includes BLEU@1 and BLEU@4. The BLEU metric is calculated by the matching degree of *N*-tuples of generated sentences and reference sentences. The METEOR value is calculated as the harmonic mean of the precision and recall rates of generated sentences. ROUGE_L mainly focuses on the calculation of the F-measure based on the longest common subsequence between the generated sentences and the reference sentences. CIDEr uses the cosine distance between the generated sentence and the reference sentence to measure the degree of match. SPICE is an indicator of assessing the diversity of an image caption. It introduces a phrase hierarchy that takes into account not only word-level matching but also phrase-level information.

### 6.4. Experimental Result

#### 6.4.1. Experimental Result on MOCS

To verify the effectiveness of our proposed model, we conduct tests on the MOCS test set and compare the results with those of other models. As presented in Table 1, we first calculate the metrics of BLEU@1, BLEU@4, METEOR, ROUGE_L, CIDEr, and SPICE based on cross-entropy loss using Equation (20), with the corresponding results shown in columns 2 through 7 of Table 1. Next, we calculate the metrics based on CIDEr-D optimization using Equation (21), and the corresponding results are shown in columns 8 through 13 of Table 1. Due to the limited amount of training data, the overall metric scores on the MOCS dataset are lower than those on the MSCOCO dataset. However, our model outperforms Up-down [18], AoANet [22], X-LAN [19], and ViTCAP [43] models in all metrics. At the CIDEr-D optimization stage, the CIDEr and SPICE metrics of our model reach 67.2% and 12.3%, respectively, which are 3.8% and 0.7% higher than those of the ViTCAP model. Our advantage is most obvious in the CIDEr metric and the SPICE metric, which fully verifies the effectiveness of our proposed model in the construction scenes. The reason for this is that the encoding part combines semantic features and style features to enable the model to fully perceive and understand the semantic style of the image. In addition, to improve the accuracy of the description results, sentence style loss is introduced to make the generated sentences more similar to the reference sentences. Furthermore, our model is trained from the original image without additional calculation of regional features of the image, which can be more convenient for application.

#### 6.4.2. Experimental Result on MSCOCO

To evaluate the effectiveness of our proposed model, we also conduct tests on the MSCOCO test set and compare the results with those of other models. Similar to the calculation of indicators on the MOCS dataset, we also utilize BLEU@1, BLEU@4, METEOR, ROUGE_L, CIDEr, and SPICE metrics to verify the model’s performance on the MSCOCO test set. We calculate the metrics based on cross-entropy loss and CIDEr-D optimization. As shown in Table 2, our research method achieves higher accuracy on the MSCOCO test set compared with the models Up-down [18], AoANet [22], X-LAN [19] and ViTCAP [43] models. Our model outperforms others in both the cross-entropy loss stage and the CIDEr-D optimization stage. Specifically, at the CIDEr-D optimization stage, the CIDEr metric reaches 137%, which is 3.9% higher than that of the ViTCAP model. SPICE achieves 24%, an improvement of 1.0% over the ViTCAP model. The images in the MSCOCO dataset depict daily life scenes. Our model demonstrates excellent performance on the MSCOCO dataset, which fully demonstrates the generalization of our model and proves that the model is also applicable to daily life scenes.

#### 6.4.3. Ablation Study

Effectiveness of the SF and SSL. To verify the effectiveness of the style feature encoding module and sentence style loss, we set an ablation experiment to remove the style feature encoder and sentence style loss, respectively. It explores the influence of the two modules on image-captioning performance. The experimental results are shown in Table 3. The first line removes the style feature encoder and sentence style loss, and the model has the worst effect. In the second line, the style feature encoder is removed, and the sentence style loss is retained. Compared with the first line, the model effect is improved, which proves the validity of the sentence style loss. In the third line, the style feature encoder is retained, the sentence style loss is removed, and the model is improved compared with the first line, which proves the effectiveness of the style feature encoding. In the fourth row, two modules are retained, and the scores of the model in six indicators are improved, which fully verifies that the two modules are compatible and have a positive effect on the experimental results.

Effectiveness of the number of blocks. We conduct a series of experiments to assess the impact of varying the number of encoder and decoder blocks. The findings, as presented in Table 4, demonstrate a notable improvement in model performance across all six metrics with an increasing number of blocks. Notably, when the number of blocks reaches four, there is no significant discernible difference in model effectiveness compared to the three-block configuration. In light of the associated escalation in model parameters and training time, coupled with the exceptional performance exhibited by the three-block model, we deem it appropriate to adopt a three-block configuration as the final setting.

Effectiveness of (S)W-MSA. Simultaneously, we conducted experiments to investigate the impact of varying window sizes and shift sizes on the performance of W-MSA and SW-MSA within the model. Considering that the feature input dimension for the encoder is 12 × 12 × 512, it can be observed that W-MSA and SW-MSA converge to MSA when the window size is set to 12. The subsequent Table 5 showcases the experimental results: the first row indicates the utilization of MSA exclusively, while the second row demonstrates the alternating incorporation of W-MSA and SW-MSA. Notably, employing a combination of W-MSA and SW-MSA yields superior outcomes compared to relying solely on MSA.

#### 6.4.4. Visualization Analysis

**Figure 6:**
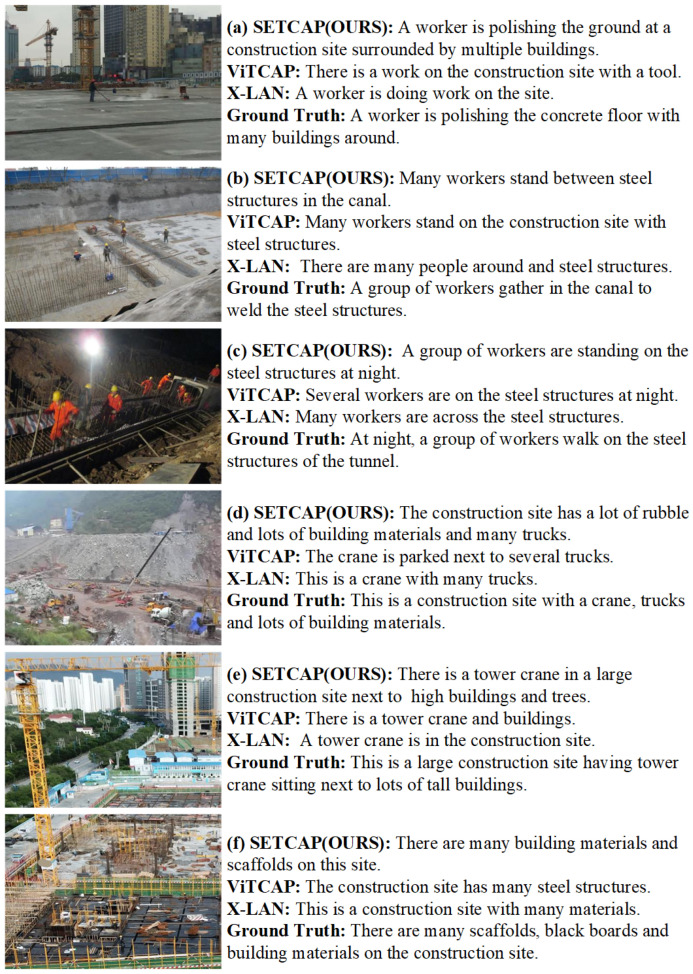
The comparison of captioning results generated by aforementioned methods on the MOCS test set.

**Figure 7:**
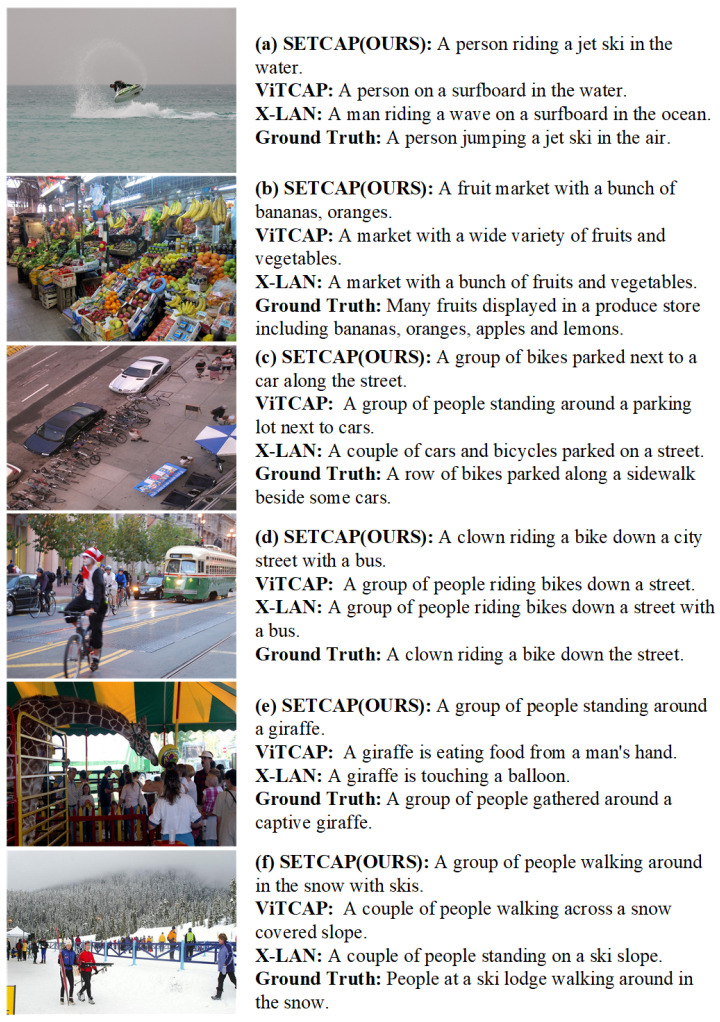
The comparison of captioning results generated by aforementioned methods on the MSCOCO test set.

To visually demonstrate the effect of the model, part of the results in the test set are visualized, as shown in Figure 6 and Figure 7. It can be seen that the proposed model has good image-captioning performance and can accurately generate text descriptions in line with the construction scenes and normal scenes.

Figure 6a–c all have specific scene activities, and generated descriptions are more detailed. Figure 6d–f are similar to an aerial view, without specific scene activities, and generated descriptions are more of an overall overview, indicating that the model can grasp the semantic style of the image. Figure 6a has a complex background, and our model can also capture the movement of workers polishing the ground, but other models do not reason about specific work activity; Figure 6b shows that our model successfully detects both the small target, “steel bars”, and the overall environment, “canal”, whereas other models fail to identify the latter. Figure 6c shows that in low-light conditions at night, our model outlines multiple workers standing at a complex construction site, providing more detailed results compared to other models; Figure 6d–f show the reasoning ability of our model under complex scenarios. Compared with other models, our model can understand the style of the image better, and the generated sentence is closer to the reference sentence.

Figure 7 demonstrates that in a normal scene, regardless of the complexity or simplicity of the background, our model exhibits superior proficiency in capturing the primary subjects and occurrences depicted within the image. Moreover, the generated sentences remarkably align with the provided reference sentences, therefore showcasing a higher degree of fidelity. Specifically, Figure 7a shows that our model generates a sentence that more accurately describes the ‘jet ski’, closer to the reference sentence. In Figure 7b, our model captures greater detail of both the environment and fruit species compared to other models. Furthermore, in Figure 7c–e, our model selects the subject and object more accurately, resulting in a sentence that is more similar to the reference sentence. Finally, in Figure 7f, our model provides a more precise description of the environment.

**Figure 8:**
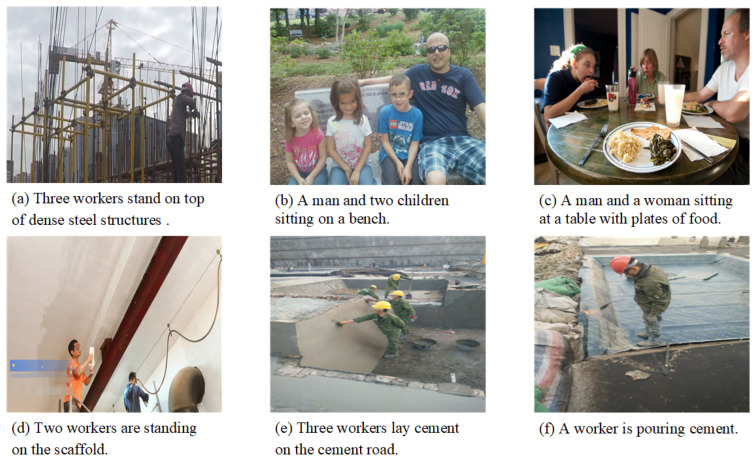
The generated failure examples by our method on dataset MOCS and MSCOCO. For the images of (**a**–**c**), they fail to detect the number of individuals exactly. The descriptions provided in (**d**–**f**) deviate from their contents. Specifically, the description in (**d**) fails to accurately represent the putty construction activity, (**e**) describes the object incorrectly, and (**f**) does not classify the construction activity correctly.

We have also analyzed selected failure examples generated by our model. The captions of images (a), (b), and (c) in Figure 8 basically describe the primary content of these images. However, they inaccurately detect the number of individuals. This is because the parameters of the Swin Transformer in our model are fixed during the training phase, which reduces the effectiveness of extracting image features. The descriptions provided in (d), (e), and (f) deviate from their respective contents. Specifically, the description in (d) fails to accurately represent the putty construction activity, (e) describes the object incorrectly, and (f) mischaracterizes the construction activity. This may be attributed to the limited scope of annotation, resulting in the model’s weak generalization ability for construction activities and tools not present in the training set.

## 7. Conclusions

Construction scenes are relatively complex compared with daily life scenes, and it is a challenging task to fully understand the semantic content of the complex construction scenes. In this paper, we first build a textual dataset based on the construction scenes and use it to train an image-captioning model. Furthermore, considering that the style of the image will affect the direction of the sentence description, two encoders are constructed. One encoder is used to encode the semantic features of the image, and the other encoder is utilized to encode the style features. Then, the image style is added to the decoder as additional information to guide the decoder to generate an appropriate description of the content. Since the annotation dataset is small, we enhanced the data of the construction scenes dataset to make the model more stable. At the same time, to make the style of the generated sentence more consistent with the training data of the construction scenes, the sentence style loss function is added to total loss. The experimental results demonstrate that the proposed method has a superior performance compared to state-of-the-art methods.

## Figures and Tables

**Table 1:** Evaluation results of our proposed model and other existing state-of-the-art models on the MOCS test set. B@N, M, R, C, and S are short for BLEU@N, METEOR, ROUGE, CIDEr, and SPICE scores. All values are reported as percentages (%). Bold text in the table indicates the best results.

Models	Cross-Entropy Loss						CIDEr-D Optimization					
	B@1	B@4	M	R	C	S	B@1	B@4	M	R	C	S
Up-down [18]	34.6	16.8	13.6	25.5	53.8	9.4	35.6	16.8	13.9	25.7	56.9	9.9
AoANet [22]	34.9	17.2	14.3	26.8	56.3	9.8	36.0	17.9	14.7	27.4	61.0	10.3
X-LAN [19]	35.6	17.7	14.5	27.2	57.4	10.1	36.7	18.3	14.9	27.8	62.1	10.8
ViTCAP [43]	36.3	17.9	14.5	27.4	58.1	11.1	37.5	18.8	14.8	28.3	63.4	11.6
SETCAP(ours)	37.2	18.3	15.2	28.4	60.5	11.3	38.4	19.3	15.4	28.8	67.2	12.3

**Table 2:** Evaluation results of our proposed model and other existing state-of-the-art models on the MSCOCO “Karpathy” test split. B@N, M, R, C, and S are short for BLEU@N, METEOR, ROUGE, CIDEr, and SPICE scores. All values are reported as percentages (%). Bold text in the table indicates the best results.

Models	Cross-Entropy Loss						CIDEr-D Optimization					
	B@1	B@4	M	R	C	S	B@1	B@4	M	R	C	S
Up-down [18]	77.2	36.2	27.0	56.4	113.5	20.3	79.8	36.3	27.7	56.9	120.1	21.4
AoANet [22]	77.4	37.2	28.4	57.5	119.8	21.3	80.2	38.9	29.2	58.8	129.8	22.4
X-LAN [19]	78.0	38.2	28.8	58.0	122.0	21.9	80.8	39.5	29.5	59.2	132.0	23.4
ViTCAP [43]	-	35.7	28.8	57.6	121.8	22.1	-	40.1	29.4	59.4	133.1	23.0
SETCAP(ours)	78.7	38.5	29.0	58.6	123.2	22.1	81.2	40.5	29.9	59.5	137.0	24.0

**Table 3:** Performance comparison with/without the style feature encoder and the sentence style loss for our proposed SETCAP. SF represents the style feature encoder, and SSL represents sentence style loss. B@N, M, R, C, and S are short for BLEU@N, METEOR, ROUGE, CIDEr, and SPICE scores. All values are reported as percentages (%).

SF	SSL	B@1	B@4	M	R	C	S
✗	✗	35.3	17.8	14.2	26.5	61.8	11.3
✗	✓	35.7	17.9	14.3	26.8	62.5	11.4
✓	✗	36.9	18.5	14.8	27.6	64.5	11.8
✓	✓	38.4	19.3	15.4	28.8	67.2	12.3

**Table 4:** The performance of different numbers of the encoder and decoder block on the MOCS dataset. B@N, M, R, C, and S are short for BLEU@N, METEOR, ROUGE, CIDEr, and SPICE scores. All values are reported as percentages (%).

Layer	B@1	B@4	M	R	C	S
1	36.4	18.1	14.7	27.3	63.6	11.6
2	37.1	18.8	15.0	28.0	65.1	12.0
3	38.3	19.3	15.4	28.8	67.2	12.3
4	38.4	19.3	15.2	28.8	67.2	12.4

**Table 5:** The influence of different window sizes and shift window sizes on the model. WS means window size, and SS means shift size. B@N, M, R, C, and S are short for BLEU@N, METEOR, ROUGE, CIDEr, and SPICE scores. All values are reported as percentages (%).

Size	B@1	B@4	M	R	C	S
WS = 12, SS = 0	37.2	18.7	15.1	27.8	65.3	11.9
WS = 6, SS = 3	38.3	19.3	15.4	28.8	67.2	12.3

## Data Availability

The MSCOCO and MOCS datasets are openly available in a public repository. They can be downloaded at https://cocodataset.org/#download, accessed on 1 April 2015, and http://www.anlab340.com/Archives/IndexArctype/index/t_id/17.html, accessed on 10 October 2020. The annotated sentences for MOCS that support the findings of this study are available from the corresponding author upon reasonable request.
